# Involvement of glutathione peroxidases in the occurrence and development of breast cancers

**DOI:** 10.1186/s12967-020-02420-x

**Published:** 2020-06-22

**Authors:** Man-Li Zhang, Hua-Tao Wu, Wen-Jia Chen, Ya Xu, Qian-Qian Ye, Jia-Xin Shen, Jing Liu

**Affiliations:** 1 Changjiang Scholar’s Laboratory/Guangdong Provincial Key Laboratory for Diagnosis and Treatment of Breast Cancer, Shantou University Medical College, Shantou, 515041 China; 2 Department of General Surgery, the First Affiliated Hospital of Shantou University Medical College, Shantou, 515041 China; 3 Department of Physiology/Cancer Research Center, Shantou University Medical College, Shantou, 515041 China; 4 Department of Hematology, the First Affiliated Hospital of Shantou University Medical College, Shantou, People’s Republic of China

**Keywords:** Glutathione peroxidase, Breast cancer, Reactive oxygen species, Occurrence

## Abstract

Glutathione peroxidases (GPxs) belong to a family of enzymes that is important in organisms; these enzymes promote hydrogen peroxide metabolism and protect cell membrane structure and function from oxidative damage. Based on the establishment and development of the theory of the pathological roles of free radicals, the role of GPxs has gradually attracted researchers’ attention, and the involvement of GPxs in the occurrence and development of malignant tumors has been shown. On the other hand, the incidence of breast cancer in increasing, and breast cancer has become the leading cause of cancer-related death in females worldwide; breast cancer is thought to be related to the increased production of reactive oxygen species, indicating the involvement of GPxs in these processes. Therefore, this article focused on the molecular mechanism and function of GPxs in the occurrence and development of breast cancer to understand their role in breast cancer and to provide a new theoretical basis for the treatment of breast cancer.

## Background

Breast cancer has become the most common cancer and the leading cause of cancer-related deaths in females worldwide, according to a status report on the global cancer burden provided by Globocan 2018 [1]. The current standard treatment for patients with breast cancer includes the combination of surgery, radiation, hormone therapy and chemotherapy drugs, such as anthracyclines, cyclophosphamide, taxanes and platinum compounds [2, 3]. As a superficial tumor, the causes of death in patients with breast cancer are reported to be due to cancer metastasis and/or relapse, which are significantly associated with the favorable tumor microenvironment [4]. However, the mechanisms of the occurrence, development, and metastasis of breast cancer are very complicated and overlap, suggesting the necessity of different therapies to treat different subtypes of breast cancer. To achieve the best treatment effect, it is necessary to adopt a customized treatment plan for patients, based on the understanding of the pathogenesis of breast cancer [5, 6].

Recent studies have shown that reactive oxygen species (ROS) are involved in the molecular mechanisms of breast cancer occurrence and development [7–9]; ROS can be regulated by glutathione peroxidases (GPxs), which are the key enzymes that maintain ROS homeostasis in vivo through the reduction of ROS [10]. Therefore, based on summaries of the research regarding the relationship between breast cancer and GPxs, this article focused on the molecular mechanism and function of different GPxs and their diverse roles in the occurrence and development of breast cancer, further clarifying the pathogenesis of breast cancer and providing a potential direction for further studies on the treatment of breast cancers.

## The GPx family and its members

**Fig. 1:**
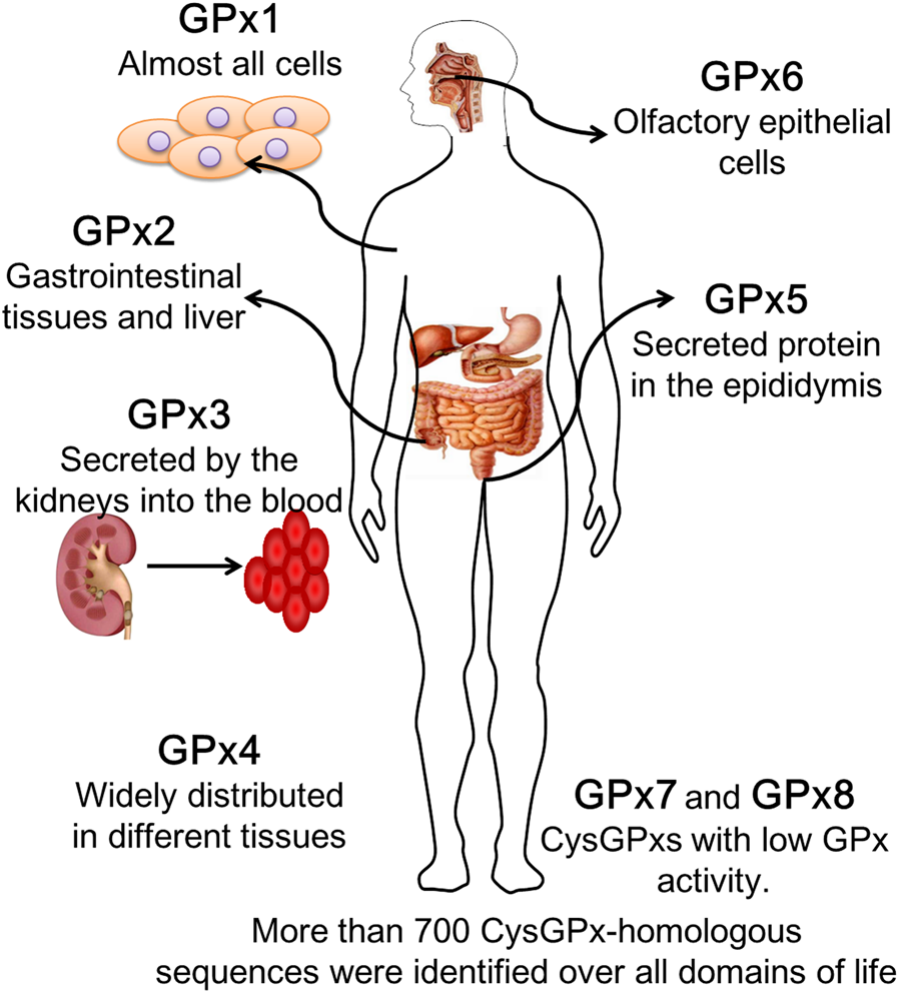
The main distribution of GPxs in human

GPxs are members of a multiple-isozyme family that catalyze the reduction of H_2_O_2_ or organic hydroperoxides to generate water or corresponding alcohols using reduced glutathione (GSH) as an electron donor [11]. Currently, 8 GPxs (GPx1–GPx8) have been identified in mammalian tissues, which exhibit the genetic, structural and dynamic differences and perform common and separate functions [12] (Fig. 1).

Among these GPxs, 5 selenium-dependent GPx isozymes have been identified (Table 1). (1) Classical GPx (GPx1) is reported to be expressed in red blood cells and liver, lung, and kidney tissues and is located in the cytosol, nucleus, and mitochondria. The antioxidant effects of GPx1 are achieved by the direct reduction of hydrogen peroxide and lipid hydroperoxides [13]. (2) Gastrointestinal GPx (GPx2) is only found in the cytosol and nucleus of the gastrointestinal tract. Because of its specific localization, GPx2 was the first GPx considered to be a barrier to the absorption of hydrogen peroxide [14]. (3) Plasma GPx (GPx3) is present in the mitochondria of several organs, such as the kidney, lung, epididymis, breast, heart, and muscle. As the main antioxidant enzyme in plasma, GPx3 is the only extracellular enzyme in the GPx family that reduces ROS products during normal metabolism or oxidative damage [15]. (4) Phospholipid GPx (PHGPx or GPx4) is found in the nucleus, cytosol, and mitochondria of various tissues. As an intracellular selenium protein, GPx4 can directly reduce the production of peroxide phospholipids in the cell membrane [16]. (5) GPx6 is expressed in the olfactory epithelium of humans and pigs [17]. However, GPx6 rarely has been studied, and its function has not been clearly defined.

**Table 1:** The types of GPxs and their proven substrates

GPxs	Types	Expressions	Locations	Oxidizing substrates	Reducing substrate	Peroxidatic residue	References
GPx1	Selenium-dependent	Red cells, liver, lung, and kidney	Cytosol, nucleus, and mitochondria	H_2_O_2_, soluble low MM hydroperoxides	GSH	Sec	[17, 83]
GPx2	Selenium-dependent	Gastrointestinal tract	Cytosol and nucleus	N.F.	N.F.	Sec	[118–120]
GPx3	Selenium-dependent	Kidney, lung, epididymis, breast, heart, and muscle	Mitochondria	H_2_O_2_, soluble low MM hydroperoxides	GSH, low rate with thioredoxin and glutaredoxin	Sec	[17, 121]
GPx4	Selenium-dependent	Various tissues	Nucleus, cytosol, and mitochondria	H_2_O_2_, small hydroperoxides, hydroperoxides in complex lipids	GSH, Dithiothreitol	Sec	[122–124]
GPx5	Non-Selenium-dependent	Epididymis	Extracellular	N.F.	N.F.	Cys	[17, 125]
GPx6	Selenium-dependent in human	Olfactory epithelium	N.A.	N.F.	N.F.	Sec (human) Cys (rats)	[17, 126]
GPx7	Non-Selenium-dependent	Preadipocytes	N.A.	H_2_O_2_	GSH, Protein disulfide isomerase	Cys	[17, 127, 128]
GPx8	Non-Selenium-dependent	Lung	Endoplasmic reticulum	H_2_O_2_	GSH	Cys	[128, 129]

*MM* molecular mass, *N.F.* not found, *N. A.* not available

Among the three non-selenium glutathione peroxidases, GPx5 is secreted from the epididymis and is thought to protect sperm from peroxide-mediated attacks during maturation. Although the active domain of GPx5 lacks selenocysteine, it retains antioxidant properties [18]. Another non-selenium glutathione peroxidase, GPx7, which lacks GPx activity, has recently been described as a novel phospholipid hydroperoxide GPx [11, 19–21]; the sequence of GPx7 encodes cysteine in its conserved catalytic motifs [22]. As a non-selenocysteine, GPx8 is a membrane protein that is detected in the endoplasmic reticulum, is present abundantly expressed in the lung, and was identified as a novel member of the GPx family [23].

In general, both the selenium-containing GPxs and non-selenium GPxs are key players in the biological environment and the development of human diseases [17]. The following section will describe the relationships and molecular mechanisms of GPxs in disease development.

## ROS are the main molecules involved in the role and mechanism of GPxs in the development of diseases

ROS, reactive oxygen-containing molecules, are the normal products of aerobic reactions in humans, such as oxidative respiration in mitochondria [24]. ROS are produced by multiple intrinsic mechanisms, and ROS come in a variety of forms, including radical (hydroxyl, superoxide, etc.) or non-radical (hydrogen peroxide, singlet oxygen, etc.) [25]. The intrinsic level of ROS in the intracellular environment plays an important role in the maintenance of cellular homeostasis in vivo; the level of ROS can be dramatically increased by external stimuli or internal stresses and can be toxic to cells [26]. To neutralize ROS, systematic biological detoxification processes are used to maintain their normal level; for example, *N*-acetylcysteine (NAC) in preadipocytes reduces adipogenesis [27]. In organisms, two systems have been developed to neutralize these compounds, including non-enzymatic and enzymatic systems. In addition to superoxide dismutases, catalases, and ascorbate peroxidases, GPxs are important components of the enzymatic systems and are the focus of this article [11] (Table 1). GPxs, which participate in the processes of antioxidant protection and detoxification, are important enzymes that directly regulate ROS levels [28]. The group of GPx enzymes, which play an irreplaceable role in the regulation of ROS homeostasis in vivo, has been thought to use GSH as a reducing agent to catalyze the reduction of H_2_O_2_ or organic peroxides to generate water or corresponding alcohols, respectively [17].

It is accepted that an excessive burden of ROS may lead to abnormal cell growth, corresponding changes in intracellular homeostasis and damage to important components of in the cell when the antioxidant defense and/or DNA repair mechanisms cannot balance the excess oxidants [29]. Oxidative stress, which results from the imbalance between the production and neutralization of ROS, is also reported to be involved in a large number of pathological states, such as Alzheimer’s disease [30], Parkinson’s disease [31], atherosclerosis [32], heart failure [33], myocardial infarction [34], and hepatic encephalopathy [35]. Importantly, oxidative damage caused by the production of ROS has been linked to the etiology of different types of human cancer [23, 36].

In the initial stage of carcinogenesis, ROS play a variety of roles, including mediating the activation of carcinogens, causing DNA damage, and interfering with responses to DNA damage [37–39]. Low levels of ROS can activate host cells in the tumor microenvironment, and promote glucose metabolism in tumors to maintain the high energy consumption of tumors by inducing mitochondrial autophagy, altering key enzymes and genes related glucose metabolism and activating signal pathways; these functions suggest a role of ROS as signaling molecules that favor tumorigenesis. However, a high level of ROS inhibits the occurrence and development of tumors by inducing apoptosis of tumor cells because of their toxic effects [40]. Recently, research on tumor treatment strategies has focused on whether the treatment that opposes or promotes oxidation exert anti-tumor effects. Accumulated evidence suggests that ROS function as second messengers in the determination of cell fate and the modification of various signaling molecules [41]. Therefore, the regulation of ROS homeostasis is crucial for maintaining the health of humans. The exploration and investigation of GPxs, as key enzymes that regulate ROS levels in humans, is of great significance to elucidate the relationship between GPxs and diseases, including tumors, to further understand the pathogenesis of diseases and to prevent ameliorate and even cure these diseases.

The diverse roles of GPxs in different kinds of tumors have been examined but remain controversial. For example, GPx1 has been reported to prevent oxidative DNA mutations, which in turn may prevent the development of tumors [42], and research shows that overexpression of GPx1 can reduce tumor growth, indicating its inhibitory effect in tumorigenesis [43]. However, the expression of GPx1 has been reported to be down-regulated in thyroid cancer [44], colorectal cancer [45, 46], and gastric cancer [47], whereas GPx1 has been demonstrated to play an oncogenic role in kidney cancer [48], pancreatic cancer [49], and laryngeal squamous cell carcinoma [50]. The abnormal expression of GPx2 was detected in different tumors, and GPx2 was up-regulated in colorectal cancer [51, 52] and down-regulated in prostate intraepithelial neoplasia [53], suggesting that GPx2 plays complex roles in tumorigenesis [23]. Currently, GPx3 is considered a new tumor-suppressor gene [23], and its hypermethylation, which is associated with the further down-regulation of GPx3, was observed in patients with Barrett’s esophagus [54], endometrial adenocarcinoma [55] and prostate cancer [56]. GPx4 is also considered a tumor suppressor because it is down-regulated in tumors [23, 43]. Current investigations have demonstrated that GPx7 has potential tumor-suppressive effects in gastric and esophageal adenocarcinoma [57–59]. Due to limited research, the roles of GPx5, GPx6, and GPx8 in tumorigenesis are still awaiting clarification.

## The role of ROS in the occurrence and development of breast cancer

The occurrence of breast cancer, the most common malignancy in females worldwide, is generally believed to be significantly associated with the accumulation of genetic damage caused by genetic alterations, which cause uncontrolled cell proliferation and/or abnormal programmed cell death, or apoptosis [60]. Excessive burden of ROS, failure of clearance mechanisms and even lack of antioxidants may lead to the accumulation of ROS and to oxidative stress [40], which are associated with genetic damage and breast cancer onset [61–65]. Sies et al. systematically reviewed the oxidative stress from the perspectives of oxidants and antioxidants, and noted that oxidants with vastly different half-lives may be observed at the site of generation or be transported to distant target sites where they exert oxidant activities in cell metabolism [63]. On the other hand, antioxidants can protect the cells from incident radiation through specialized pigments and control the levels of reactive species which otherwise might cause a cascade of reactions and lead to the generation of pathological oxidants [63].

Considering the molecular mechanism of ROS in the mammary gland, it appears that the oxidative state is involved in the initial occurrence and development of breast cancer through interference with breast cancer stem cells (CSCs) [66]. Malins et al. found that oxidative stress changed the redox potential of the breast, leading to drastic changes in the DNA base lesions, which are conducive to oxidative conditions and breast cancer formation [64]. Interestingly, a positive feedback loop between miR526b/655 and oxidative stress was identified in breast cancer, and the loop promotes tumor growth and metastasis [67]. Rodrgues et al. analyzed the lipid profile and aquaporin expression under conditions of oxidative stress in different types of breast cancer cells and predicted the subsequent metabolic reprogramming of cancer cells and adaption to stress and resistance to therapies [68].

The development of drug resistance is a challenge for the use of adjuvant therapies in breast cancer patients [69], and studies suggest that ROS may play an important role in the production of drug-resistant cells [70]. Zhong et al. demonstrated that oxidative stress and H_2_O_2_ treatment led to a marked increase in senescence-associated β-galactosidase activity but only to minimal apoptotic cell death in CSCs, suggesting that ROS triggered the induction of senescence and exhibited therapeutic potential in the eradication of drug-resistant CSCs [8]. However, the increase in ROSs induced by drugs seems to play a different role compared to the role of ROS originally generated in malignant tumors. In the treatment of breast cancer, anti-estrogen tamoxifen, the drug most often used for the long-term treatment of early breast cancer, can induce apoptosis in many cells, and this effect may be mediated by ROS generation in the mitochondria of breast cancer cells [71].

## The role of GPxs in breast cancer

As mentioned above, ROS is closely related to the molecular mechanism of breast cancer progression, and ROS can be directly regulated by GPxs, which eliminate organic peroxides at the expense of GSH [10]. The following section will illustrate the relationship between different GPxs and breast cancer and their underlying molecular mechanisms to provide opportunities for further investigation and research and to benefit patients with breast cancer (Table 2).

**Table 2:** The expression, function and potential mechanism of GPxs in breast cancers

GPxs	Location	Main findings in breast cancers	References
GPx1	3p21.31	TFAP2C regulates GPx1 promoter through an AP-2 regulatory region	[81]
		GPx1 polymorphism in modifying stress response	[82]
		Decreased GPx1 expression	[83]
		Loss of heterozygosity and allelic differences of GPx1	[88]
		Pro198Leu-associated decreased GPx1 activity with high breast cancer risk	[89, 92]
		The Leu198Leu genotype of GPx-1	[91]
		Regulate the sensitivity to doxorubicin	[93]
GPx2	14q23.3	Upregulated in breast cancer cells	[19]
		Overexpression in rat breast cancer	[98]
		Highly regulated by retinoic acid	[94]
GPx3	5q33.1	Downregulated in aggressive phenotype of breast cancer	[103]
		An independent predictive marker for local recurrence of early-stage invasive cancer patients	[104]
GPx4	19p13.3	Downregulated in breast cancer cells	[19]
		Predict poor prognosis of invasive ductal breast carcinoma	[106]
		Impaired GPx4 expression in peripheral blood monocytes as a biomarker for increased breast cancer risk	[111]
GPx5	6p22.1	Downregulated in breast cancer cells	[19]
GPx6	6p22.1	Downregulated in breast cancer cells	[19]
GPx7	1p32.3	Downregulated in breast cancer cells	[19]
GPx8	5q11.2	Not available	

### GPx1

The *GPx1* gene, which encodes the first identified selenoprotein, is located on chromosome 3p21 [72, 73]. Previous research has shown that GPx1 is an effective antioxidant enzyme that cannot be replaced by any other selenoprotein to protect against generalized oxidative stress [74–76]. When cancerous cells are generated, the expression of GPx1 is abnormal, causing intracellular ROS dysfunction [77, 78]. Due to its antioxidant properties, GPx1 is thought to be highly effective in preventing the ROS-mediated initiation of cancer.

In breast cancer, transcription factor AP-2 gamma (TFAP2C) is an important transcription factor that regulates estrogen receptor-alpha (ERα) and c-ErbB2/HER2 (Her2), which are involved in the establishment of the gene expression pattern observed in different clinical phenotypes of breast cancer [79, 80]. Interestingly, Kulak et al. confirmed that TFAP2C regulated GPx1 expression by directly binding to the GPx1 promoter, which contains an AP-2 regulatory region, and the methylation of the CpG island in the GPx1 promoter region prevented the binding of TFAP2C by encompassing the AP-2 regulatory region [81]. As expected, compared to those healthy control subjects, the tissues of breast cancer patients exhibit significantly increased lipid peroxidation, which is dependent on functional polymorphism of GPx1 [82]. However, compared to that in non-malignant tissues, the expression level of GPx1 in tumor tissues was significantly decreased by 7.4% [83].

The controversial expression pattern of GPx1 in breast cancers requires further investigation to determine its molecular functions. Artificial overexpression of GPx1 showed an increased capacity to rescue breast cancer cells from the cell cycle arrest caused by hyperoxic stress, indicating the involvement of both peroxide-derived free radicals and nonperoxide-derived species during the deleterious process [84]. Gouaze et al. found that increased GPx1 levels significantly increased the resistance of breast cancer T47D cells to doxorubicin partially by interfering with the activation of the sphingomyelin-ceramide pathway [85]. Although such research is limited, the overexpression of GPx1 in breast cancer is thought to promote the occurrence and development of breast cancer.

Hence, due to the controversial evidence, exploring the molecular regulation of GPx1 expression and activity is very important for understanding the mechanisms of cancers. Frequent loss of heterozygosity (LOH) on chromosome 3p in lung tumors was associated with low GPx1 enzyme activities, which may affect the prognosis of lung cancer patients [86]. Moscow et al. first reported a polymorphism in GPx1, namely, a substitution at codon 198 of either proline (Pro) or leucine (Leu), Pro198Leu (SNP: 1050450), in lung cancer [87]. In a Macedonian population, the GPx1 Pro198Leu genotype showed an overall protective effect on prostate cancer risk, and erythrocyte GPx1 activities were significantly decreased in prostate cancer patients compared with controls [73]. Similarly, in breast cancer, LOH at this locus occurred in approximately 36% of breast cancer tissues, and GPx1 with a leucine-containing allele exhibited lower GPx1 activities in response to stimulation than GPx1 a proline-containing allele [88]. Ravn-Haren et al. showed similar results, namely, that Pro198Leu-associated GPx1 activities were decreased in breast cancer but were associated with increased breast cancer risk among Danish females [89]. In addition to the Leu198Leu genotype in the GPx1 gene, the Ala16Ala genotype in the manganese superoxide dismutase (*MnSOD*) gene, the most significant enzyme that protects against ROS in the human body [90], was also associated with an increased risk of breast cancer [91]. It is suggested that Leu carriers with the Pro198Leu *GPx1* allele had a 1.9-fold increased risk of non-ductal breast cancer and a 2.6-fold increased risk of developing grade 3 ductal tumors [92]. These results indicated that the Pro198Leu polymorphism in *GPx1* and the associated LOH are factors of significance in breast cancer development.

**Fig. 2:**
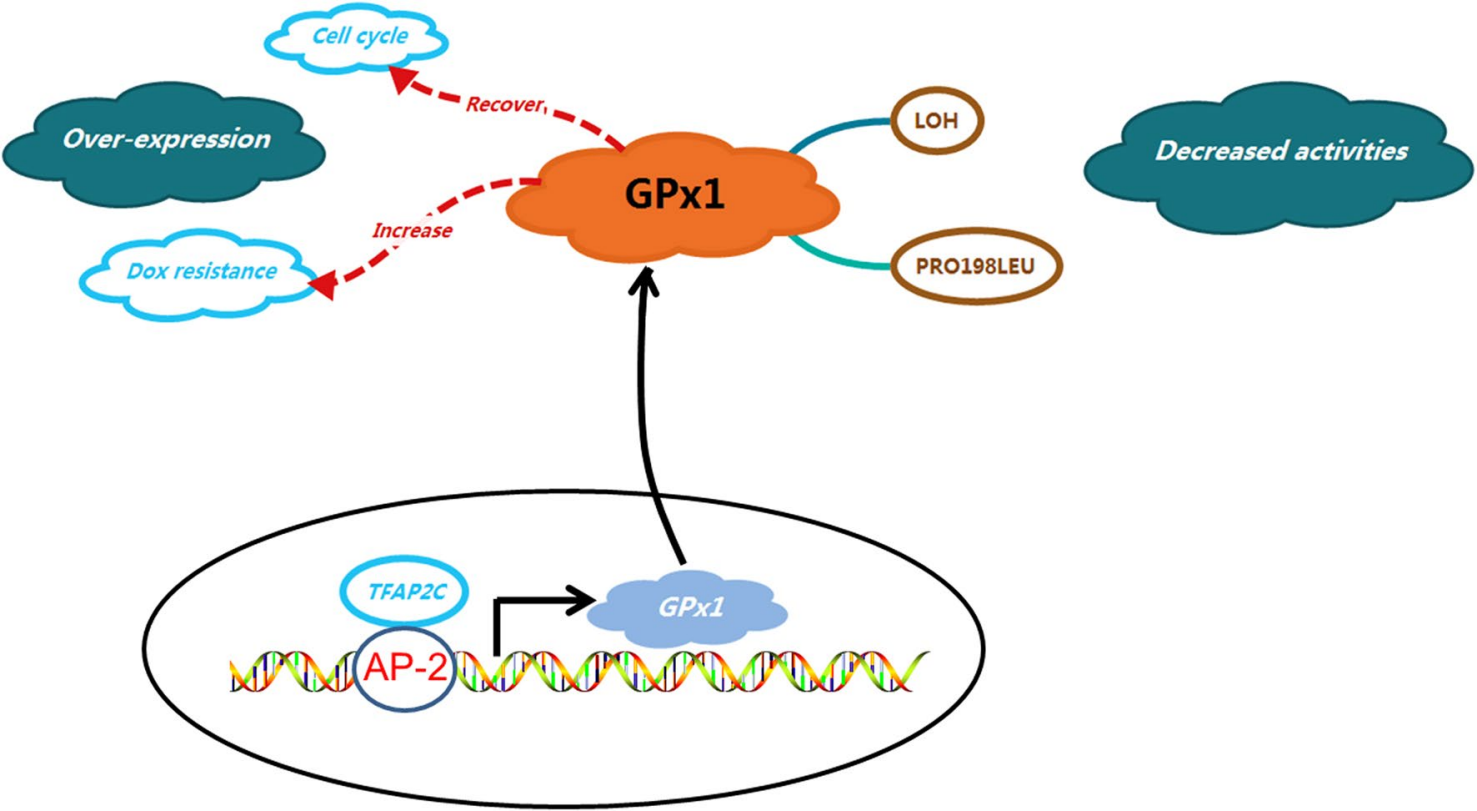
The reported function and mechanism of GPx1. *TFAP2C* transcription factor AP-2 gamma, *AP-2* AP-2 regulatory region, *LOH* loss of heterozygosity, *Dox* doxorubicin

Regarding treatment strategies, it is suggested that the marked decrease in GPx1 expression may be the major mechanism of tumor sensitization to anthracyclines [93] and that GPx1 can partially inhibit doxorubicin-induced cell death-related signaling in breast cancer by interfering with the activation of the sphingomyelin-ceramide pathway [85]; these findings suggest that tumor regression in response to chemotherapy was correlated with the inhibition of GPx1 activity and confirmed the role of GPx1 in the occurrence and development of breast cancer (Fig. 2).

### GPx2

The selenium-dependent glutathione peroxidase GPx-GI encoded by GPx2 is highly expressed in gastrointestinal epithelial cells and occasionally in breast tissue [94]. In addition to normal tissues, it has been reported that GPx2 is upregulated in a variety of tumor cells [76, 95] and is associated with tumor cell proliferation and poor prognosis of patients [96, 97].

In breast cancer, GPx2 is commonly overexpressed in the mammary carcinomas of mouse models of breast cancer induced by carcinogens, which is consistent with its up-regulation in human breast cancer [19]. As expected, the inhibition of GPx2 expression by siRNA led to significant growth inhibition in both rat and human breast cancer cell lines [19, 98]. Chu et al. confirmed that in MCF-7 cells, which a low aggressive breast cancer cell line, the expression of GPx2 can be substantially up-regulated by retinoic acid [94]. Due to its potential function in the occurrence and development of breast cancer, GPx2 is suggested as a potential target for the prevention and treatment of breast cancer. However, the current research on the relationship between GPx2 and breast cancer is limited and there are still many gaps in the research regarding its regulatory mechanisms.

### GPx3

As mentioned above, elevated levels of ROS play a crucial role in the progression of breast cancer [99]. As the only extracellular enzyme in the GPx family, GPx3 is an essential enzyme that is responsible for removing ROS products during normal metabolism or oxidative damage in healthy tissues [76]. Lee et al. observed a high frequency of promoter hypermethylation and progressive loss of GPx3 expression in Barrett’s esophagus and its associated lesions, and these authors also confirmed the known function of GPx3 as a potent antioxidant [54]. In human thyroid cancer, GPx3 is frequently methylated, and the expression of GPx3 is regulated by methylation of the promoter region, and this methylation is related to tumor size and lymph node metastasis through the inhibition of Wnt/β-catenin signaling [100]. The methylation of the GPx3 promoter was also observed in hepatocellular carcinoma (HCC) tissue [101] and gastric carcinoma [102], possibly leading to subsequent carcinogenesis and cancer cell progression. These findings suggest that epigenetic inactivation and regulation of the GPx3 pathway may be critical in the development and progression of different types of cancers.

Consistently, the GPx3 levels were reported to be down-regulated in aggressive inflammatory breast cancer (IBC) carcinoma tissues compared to non-IBC tissues, and this down-regulation was associated with hypermethylation of the GPx3 promoter [103]. In addition, a low level of GPx3 is an independent predictive marker of the local recurrence of early-stage invasive breast cancer in patients undergoing breast-conserving surgery and radiotherapy, regardless of the patient’s clinicopathological parameters [104]; this observation suggests that inactivation of the GPx3 gene by hypermethylation of the promoter may contribute to breast cancer progression.

### GPx4

GPx4 was discovered in 1982 by Ursini et al. [105], and GPx4 is the only known enzyme able to reduce lipid peroxides bound to cell membranes [106, 107]. In contrast to other GPxs, GPx4 is the only one that reduces the hydroperoxides of lipoproteins and complex lipids, such as cholesterol, cholesterol esters, and phospholipids [108], to protect mitochondrial ATP generation from oxidative damage [109]. Recently, GPx4 was predicted to be a specific target for new pharmacological therapies aimed at activating or inhibiting cell death in cancer or degenerative diseases [110].

Interestingly, GPx4 expression was down-regulated in breast cancer cells compared with normal breast cells [19]. A recent study showed a strong negative correlation between breast tumor grade progression and GPx4 expression in breast cancer tissues, and its downregulation may be related to the poor prognosis of patients with invasive ductal carcinoma of the breast [106]. In addition, impaired GPx4 expression in peripheral blood monocytes was shown to be a biomarker of increased risk of breast cancer [111]. However, the specific mechanism of GPx4 in the occurrence of breast cancer has not been elucidated and requires further study.

### Other GPxs

As studies of the functions of GPx5, GPx6 and GPx7 in the development of breast cancer are limited, it is difficult to determine their underlying mechanisms. Rusolo et al. systemically examined the expression of GPxs in breast cancer cell lines and found that GPx5 and GPx7 were down-regulated in the human breast cancer MCF-7 and MDA-MB-231 cell lines and GPx6 was also down-regulated in MDA-MB-231cells compared with healthy breast MCF-10A cells [19]. However, relevant research on their roles in breast cancer and the regulatory mechanisms are still lacking and needs to be supplemented. The expression pattern and function of GPx8 in breast cancer have not been reported.

## Therapeutic implications

Based on the role of ROS in breast cancer, several reagents have been reported to be potential therapeutic methods for patients with breast cancer. Methanolic extract of corn silk, which induces apoptosis in breast cancer cells was proposed to be a novel anti-tumor agent that acts by increasing ROS production and decreasing GSH levels in a dose-dependent manner [112]. De et al. reported the therapeutic potential of another natural quinazoline derivative that promoted oxidative stress and increased ROS production [113]. George et al. proposed Rubus bioactive compounds as another reagent to induce apoptotic cell death in human breast cancer cells [114]. In triple-negative breast cancer (TNBC) cells treated with isorhamnetin (IH) and chloroquine (CQ), ROS induced Drp1-dependent mitochondrial fission and apoptosis by mediating the activation and mitochondrial translocation of CaMKII; this study suggested IH as a novel chemotherapeutic agent [115].

As an important group of antioxidative enzymes, the normal levels of GPxs help to control the level of reactive species. The identification of ways to improve the expression of GPxs in malignant tumors, especially in breast cancers, is further research direction. However, to date, the methods used to increase GPx expression are mainly the artificial over-expression of GPxs through transfections or infections. After confirming the clinical significance and potential therapeutic role of GPx3 in tumor recurrence after liver transplantation [116], Qi et al. constructed and verified a delivery system by composed of mesenchymal stem cells derived from human induced pluripotent stem cells (hiPSC-MSCs) to increase the expression level of GPx3 in vivo [117]. The engineered hiPSC-MSCs successfully delivered GPx3 to the liver and ameliorated hepatic injury by inhibiting hepatic senescence [117]; this result provides potential therapeutic strategies and prospects for breast cancer treatment. Based on the review of key role of ROS in the occurrence and development of breast cancer, it is inspired that the changed expression level of GPxs may achieve the therapeutic purpose after determining its clinical significance in breast cancer. For example, the inactivation of GPx3 may contribute to the progression of breast cancer [103, 104]. So the delivery of GPx3 to breast tumor tissues through hiPSC-MSCs or other methods may promote its expression, and reduce the influence of ROS on the progression of breast cancer accordingly, finally achieve the goal of treating breast cancer. Although there is no corresponding research report at present, this method undoubtedly provides a new idea and strategy for the treatment of breast cancer.

## Conclusion

Breast cancer is a serious threat to the health of women around the world, and the mechanism of its occurrence and treatment is of great significance. The identification of the relationship between GPxs and breast cancer is valuable for the discovery of potential therapeutic targets for breast cancer. A greater understanding of the pathogenesis of breast cancer will increase the chances of identifying a cure. At present, there has been substantial research on the relationship between GPxs and breast cancer, but the research is still very limited. This review provides a systematic and clear summary of the relationship between GPxs and breast cancer to elucidate the role of GPxs in the pathogenesis of breast cancer and to provide a clear direction. There is a large gap waiting to be filled by subsequent researchers to yield better therapeutic targets and methods to cure breast cancer, and the role of GPxs in breast cancer should be studied as an extremely important issue. Additional studies of GPxs in breast cancer may 1 day lead to a cure for this disease that is so devastating to women’s lives and health.

## Data Availability

Not applicable.

## Abbreviations

CQ: Chloroquine
CSCs: Cancer stem cells
ERα: Estrogen receptor-alpha
GPxs: Glutathione peroxidases
GSH: Glutathione
HCC: Hepatocellular carcinoma
hiPSC-MSCs: Mesenchymal stem cells drived from human induced pluripotent stem cells
IBC: Inflammatory breast cancer
IH: Isorhamnetin
LOH: Loss of heterozygosity
MnSOD: Manganese superoxide dismutase
NAC: *N*-acetylcysteine
PHGPx: Phospholipid GPx
ROS: Reactive oxygen species
TNBC: Triple-negative breast cancer
TFAP2C: Transcription factor AP-2 gamma
